# Bridging cardiology and oncology in the era of precision medicine

**DOI:** 10.1007/s00395-025-01097-x

**Published:** 2025-02-03

**Authors:** Tienush Rassaf

**Affiliations:** Department of Cardiology and Vascular Medicine, University Hospital Essen, Essen, Germany

Cardio-oncology has emerged as a critical interdisciplinary field, addressing the intersection of cardiovascular diseases and cancer, two of the leading causes of morbidity and mortality worldwide. This special issue of *Basic Research in Cardiology* brings together a series of state-of-the-art manuscripts, each providing unique insights into the challenges, mechanisms, and therapeutic opportunities in cardio-oncology.

The ever-expanding arsenal of anticancer therapies has transformed cancer care, but at the cost of considerable cardiotoxicity. Anthracyclines, long considered a cornerstone in cancer treatment, remain notorious for their dose-dependent cardiotoxic effects. Manuscripts like “Characterization of anthracycline-induced cardiotoxicity by diffusion tensor magnetic resonance imaging” and “Cardioprotection strategies for anthracycline cardiotoxicity” moved one step further in advanced diagnostic and protective strategies with a state-of-the-art review of imaging and molecular cardioprotection. In this same context, the contribution entitled “The role and mechanism of epigenetics in anticancer drug-induced cardiotoxicity” has provided insights on the epigenetic regulation of the cardiotoxic pathways and therefore new avenues for therapeutic targeting.

The rise of immune checkpoint inhibitors (ICI) has revolutionized oncology, but these agents are also associated with cardiovascular toxicities that demand urgent attention. Manuscripts in this special issue, such as “Molecular fingerprints of cardiovascular toxicities of immune checkpoint inhibitors” and “Preclinical models of cardiotoxicity from immune checkpoint inhibitor therapy,” underscore the importance of immune-mediated mechanisms underlying ICI-related cardiotoxicity. These studies illustrate how immune modulation affects cardiac function in preclinical and translational models. Moreover, “Early microvascular coronary endothelial dysfunction precedes pembrolizumab-induced cardiotoxicity” discusses the possibility of preventive strategies, such as high-dose atorvastatin, in reducing such risks.

Various manuscripts discuss metabolic interaction between cancer and cardiovascular health. “Changes in tumor and cardiac metabolism upon immune checkpoint inhibition” and “Sodium–glucose cotransporter 2 inhibitors and the cancer patient: from diabetes to cardioprotection and beyond” illustrate how changes in metabolism due to either cancer or its treatment affect the heart. Knowledge of such metabolic alterations offers the opportunity for specific interventions to maintain cardiac health while fighting tumors.

Exercise remains central to both primary and secondary prevention in cardiovascular diseases. Manuscripts like “Exercise, cancer, and the cardiovascular system: clinical effects and mechanistic insights” and “Cardioprotection of voluntary exercise against breast cancer-induced cardiac injury via STAT3” present the protective role of exercise against cancer-related cardiac injury. These findings emphasize the value of incorporating structured physical activity within cancer care to promote overall health and reduce heightened cardiovascular risk.

A recurring theme in cardio-oncology has been the influence of sex, age, and comorbidities on treatment outcomes. For instance, “Effects of sex and obesity on immune checkpoint inhibition-related cardiac systolic dysfunction in aged mice” underlines personalized approaches to mitigate cardiovascular risks in vulnerable populations. Understanding such nuances will be quite necessary as we move toward precision medicine.

## Future directions in cardio-oncology

The wide-ranging topics represented in this special issue illustrate the complexity and the multidisciplinary nature of cardio-oncology. These manuscripts collectively chart the pathway from molecular mechanisms to clinical strategies. Important future research directions involve:

**- Biomarker development:** clearly, there will be a need for robust biomarkers that can allow early detection and monitoring of cardiotoxicity with various forms of anticancer therapy.

- **Novel therapeutics:** there is an increasing need for the development of cardioprotective agents tailored to novel cancer treatment modalities, including ICIs.
- **Preclinical models:** better preclinical models will help in translating basic science findings into the clinic.
- **Lifestyle interventions:** more studies are needed on the integration of exercise and dietary interventions into cancer therapy in terms of comprehensive cardiovascular risk reduction.

This special issue of the journal underscores the need for a collaborative approach in which oncologists, cardiologists, and researchers join forces to address the dual burden of cancer and cardiovascular disease. As our knowledge increases, so does our ability to develop appropriate, multidisciplinary interventions that improve outcomes in patients facing these intertwined challenges.

In conclusion, cardio-oncology is a challenge and an opportunity. By leveraging cutting-edge science and fostering collaboration across disciplines, we can ensure that advances in cancer therapy are not achieved at the expense of cardiovascular health. I extend my gratitude to the authors and reviewers for their contributions to this timely and impactful special issue of *Basic Research in Cardiology*.

